# Association between vaginal washing and vaginal bacterial concentrations

**DOI:** 10.1371/journal.pone.0210825

**Published:** 2019-01-24

**Authors:** Michelle C. Sabo, Jennifer E. Balkus, Barbra A. Richardson, Sujatha Srinivasan, Joshua Kimani, Omu Anzala, Jane Schwebke, Tina L. Feidler, David N. Fredricks, R. Scott McClelland

**Affiliations:** 1 Department of Medicine, University of Washington, Seattle, WA, United States of America; 2 Department of Global Health, University of Washington, Seattle, WA, United States of America; 3 Department of Epidemiology, University of Washington, Seattle, WA, United States of America; 4 Vaccine and Infectious Disease Division, Fred Hutchinson Cancer Research Center, Seattle, WA, United States of America; 5 Department of Biostatistics, University of Washington, Seattle, WA, United States of America; 6 Institute for Tropical and Infectious Diseases, University of Nairobi, Nairobi, Kenya; 7 Department of Medical Microbiology, University of Nairobi, Nairobi, Kenya; 8 Division of Infectious Disease, University of Alabama at Birmingham, Birmingham, AL, United States of America; University of Cape Town, SOUTH AFRICA

## Abstract

Vaginal washing is a common practice associated with adverse outcomes including bacterial vaginosis (BV) and HIV infection. Prior studies have not examined the associations between vaginal washing and individual vaginal bacteria, or whether these associations are independent of the effect of vaginal washing on BV. The purpose of this study was to characterize the association between vaginal washing and the presence and concentrations of vaginal bacteria associated with optimal and sub-optimal vaginal states. The analysis utilized data from participants in the placebo arm of the Preventing Vaginal Infections trial, which enrolled HIV-uninfected women from the United States and Kenya. Detection of bacterial taxa associated with BV was compared between visits with versus without reported vaginal washing. The effect of vaginal washing on a number of vaginal bacteria differed substantially (p<0.05) between the US and Kenya, so results were stratified by country. In US women, vaginal washing was associated with a significantly higher likelihood of detection of BV associated bacterium 1 (BVAB1) (relative risk [RR] 1.55, 95% confidence interval [CI] 1.15–2.09, p = 0.004), BVAB2 (RR 1.99, 95%CI 1.46–2.71, p<0.001), *Mageeibacillus indolicus* (RR 2.08, 95%CI 1.46–2.96, p<0.001), *Atopobium vaginae* (RR 1.34, 95%CI 1.13–1.59, p = 0.001), *Leptotrichia/Sneathia* species (RR 1.66, 95% CI 1.33–2.09, p<0.001), *Megasphaera* species (RR 1.78, 95%CI 1.34–2.37, p<0.001) and *Gardnerella vaginalis* (RR 1.08, 95%CI 1.01–1.16, p = 0.02). No significant association between vaginal washing and bacterial detection was found in Kenyan women. Adjustment for bacterial vaginosis diagnosed by Gram stain did not alter these results. This study provides evidence that the association between vaginal washing and detection of individual bacterial taxa can vary regionally. For some vaginal bacteria, the association with vaginal washing may be independent of the effect on Gram stain detection of BV. Larger prospective studies in diverse geographic settings should explore whether eliminating vaginal washing impacts the presence and concentrations of key vaginal bacteria.

## Introduction

Vaginal washing is common worldwide, and is motivated by perceptions of cleanliness and hygiene [1–3]. Methods of vaginal washing vary greatly across different regions. For example, US women are more likely to douche (using a jet or stream of water) a few times a month using commercial products, whereas African women are more likely to use a hand or piece of cloth to wash inside the vagina as often as daily, but using water, household remedies, or soaps [1, 4, 5]. In contrast to the perceived benefits, vaginal washing has been associated with increased risk of bacterial vaginosis (BV) and human immunodeficiency virus (HIV) acquisition [6–11].

It has been hypothesized that adverse outcomes associated with vaginal washing are mediated by changes in vaginal microbiota. The vaginal microbiome likely exists on a continuum between an “optimal” state, with high concentrations of *Lactobacillus* species, and “sub-optimal” states, characterized by increased bacterial species diversity and high concentrations of anaerobic species. Advances in molecular methods have allowed for identification of sub-optimal bacteria associated with BV, adverse pregnancy outcomes, and HIV acquisition [12–16]. This study sought to address two questions. First, an analysis was conducted to examine whether the associations between vaginal washing and the presence and concentrations of vaginal bacteria differed significantly in women from the US versus Kenya, two geographically distinct regions with different vaginal washing practices. A second analysis evaluated the associations between vaginal washing and ten bacterial taxa known to be associated with vaginal health (*Lactobacillus crispatus*, *L*. *jensenii*, and *L*. *iners)* or BV (bacterial vaginosis-associated bacterium type 1 [BVAB1], BVAB2, *Mageeibacillus indolicus*, *Atopobium vaginae*, *Leptotrichia/Sneathia* species, *Megasphaera* species and *Gardnerella vaginalis*) in US and Kenyan women [14].

## Materials and methods

### Study populations and procedures

An analysis was performed using data collected from participants enrolled in the placebo arm of the Preventing Vaginal Infections (PVI) trial, a randomized, placebo controlled trial assessing monthly intravaginal metronidazole plus miconazole for reducing BV and vulvovaginal candidiasis. Detailed study procedures have been published [17]. In brief, 234 women ages 18–45 from the US and Kenya were enrolled from May 2011 to August 2012. Eligible participants had one or more vaginal infections at screening (BV, vulvovaginal candidiasis, or *Trichomonas vaginalis* [TV]). Women with symptomatic infections or TV were treated. Participants were instructed to return for enrollment 7–28 days after the screening visit. Because the intervention had a significant impact on BV, only data from women in the placebo arm were included in the present analysis [18, 19]. At enrollment, participants completed a face-to-face interview to provide demographic, clinical, and behavioral data. Vaginal washing data were updated at monthly follow-up visits. A pelvic speculum examination was performed at enrollment and at months 2, 4, 6, 8, 10 and 12. Vaginal fluid samples were collected and stored as previously described [18]. In the placebo arm of the trial, suppositories containing vehicle (Whitespol S55) were dispensed at monthly study visits. Women were instructed to use the suppositories nightly for five consecutive nights each month, and were encouraged to begin use of the study product on the day it was dispensed. Thus, samples were collected approximately 23 days after use of the placebo product, assuming 28-day visit intervals. Written, informed consent was obtained at enrollment for both trial participation and storage and future testing of biological specimens. Approval for the trial was obtained from the human subject’s research committees from the University of Washington (Seattle), the University of Alabama at Birmingham, and Kenyatta National Hospital (Nairobi). The PVI trial was registered at ClinicalTrials.gov (NCT01230814; http://clinicaltrials.gov).

### Laboratory procedures

Vaginal samples were transported on dry ice to the Fred Hutchinson Cancer Research Center in Seattle, WA. Extraction of DNA and qPCR assays targeting the 16S rRNA gene from the following taxa were performed as previously described: bacterial vaginosis-associated bacterium type 1 (BVAB1), BVAB2, *Mageeibacillus indolicus*, *Atopobium vaginae*, *Leptotrichia/Sneathia* species, vaginal *Megasphaera* species, *Gardnerella vaginalis*, *Lactobacillus crispatus*, *L*. *jensenii*, and *L*. *iners* [13, 19–21]. No significant variation was noted when samples were run as duplicates or singlets by qPCR (variance 7%), thus all samples were run as singlets. Controls included extraction from sham swabs to assess for contamination, evaluation for PCR inhibitors using an exogenous jellyfish amplification control, and measurement of 16S rRNA gene copies for each sample [19, 21, 22].

### Statistical analysis

Treatment at the screening visit, which could impact the vaginal microbiota at the subsequent enrollment visit, was not captured in the trial dataset [18, 19]. Therefore, bacterial qPCR data from enrollment visits were excluded from these analyses. Clinical data from enrollment visits potentially altered by antibiotic use (Nugents score, Amsel’s criteria, diagnosis of cervicitis or vulvovaginitis) were also excluded. All other baseline data, including clinical and demographic characteristics, were from the enrollment visit. Differences in baseline characteristics between US and Kenyan participants were analyzed using Fisher’s exact tests for categorical variables and Mann-Whitney U tests for continuous variables. The primary exposure, vaginal washing, was defined as washing beyond the vaginal introitus. Two types of outcome were evaluated. First, detection of bacterial taxa was defined as detection of bacteria at a concentration above the lower limit of detection (LLD) using highly sensitive qPCR assays [13, 19–21]. Second, previously described receiver operating curve (ROC) cutoffs were used to characterize concentrations of bacteria above versus below threshold values that optimize prediction of BV in this dataset [18]. Interaction terms that included country and vaginal washing were used to assess effect modification by country. Based on a significant (P<0.05) effect modification for two bacterial taxa, further analyses for all bacterial taxa were stratified by country. Generalized estimating equations with a Poisson link were used to generate relative risks (RR) and 95% confidence intervals (CI) for detection of bacterial species above the LLD and ROC cut-offs at visits where vaginal washing was reported compared to visits with no vaginal washing. Adjusted analyses were performed to control for age, unprotected sex, HSV-2 serostatus, and menstrual cycle (modeled as a categorical variable), which were selected *a priori* based on known or suspected confounding relationships with vaginal washing and vaginal bacteria [9, 23]. Categories used to define menstrual phase included: i) follicular phase (0–14 days since the start of the most recent menstrual period), ii) luteal phase (15–28 days since the start of the most recent menstrual period), iii) >28 days since the start of the most recent menstrual period or, iv) amenorrheic (no menstrual period for >3 months). Analyses were conducted using IBM SPSS Version 24. Binomial 95% confidence intervals for bacterial detection above the LLD and ROC cutoffs were calculated using online statistical software [24].

## Results

Of 116 women in the placebo arm of the trial, five did not consent to future testing of specimens. The remaining 111 women contributed 630 follow-up visits at which specimens were collected. The majority of participants (91/111, 82%) came to all 6 specimen collection follow-up visits. Baseline characteristics of the study population are presented in Table 1. By design, the study included 26 US and 85 Kenyan women. Their median age was 29 (Interquartile Range (IQR) 23–34), and 107/111 (96.4%) reported black race. At baseline, 17/111 (15.3%) participants reported vaginal washing, and all were from Kenya. Over the course of the study, 7/26 (26.9%) US women reported vaginal washing at 16 of the 149 study visits contributed by US women (10.7%). All 7 US women who reported vaginal washing were of black race. A total of 24/85 (28.2%) Kenyan women reported vaginal washing, and contributed 77/481 (16.0%) vaginal washing visits. Vaginal washing methods varied by country. Kenyan women reported washing with water alone (37/77, 48%), soap and water (39/77, 50.6%), or salt and water (1/77, 1.3%) at vaginal washing visits. In contrast, US women were more likely to report use of commercial products (8/16, 50%) or vinegar and water (2/16, 12.5%) compared to water alone (1/16 6.3%) or soap and water (5/16, 31.3%). Additional baseline differences between US and Kenyan women included reported frequency of unprotected sex in the past week (11/26, 42.3% versus 28/85, 32.9%) and exchange of sex for payment (1/26, 1.6% versus 60/85, 70.6%).

**Table 1:** Baseline characteristics of 111 US and Kenyan women.

Age	All participants (N = 111)	US (N = 26)	Kenya (N = 85)	p-value
18–25	**38 (34.2%)**	**11 (42.3%)**	**27 (31.8%)**	**0.03**
26–35	**53 (47.7%)**	**7 (26.9%)**	**46 (54.1%)**	
36–45	**20 (18.0%)**	**8 (30.8%)**	**12 (14.1%)**	
Race^1^				
Black	**107 (96.4%)**	**22 (84.6%)**	**85 (100.0%)**	**<0.001**
White	**4 (3.6%)**	**4 (15.4%)**	**0 (0.0%)**	
Hispanic	**1 (0.9%)**	**1 (3/8%)**	**0 (0.0%)**	
Marital status				
Married	**30 (27.0%)**	**7 (26.9%)**	**23 (27.1%)**	**0.006**
Never married	**33 (29.7%)**	**14 (53.8%)**	**19 (22.4%)**	
Separated/Divorced	**43 (38.7%)**	**5 (19.2%)**	**38 (44.7%)**	
Widowed	**5 (4.5%)**	**0 (0.0%)**	**5 (5.9%)**	
Contraceptive use				
None	**16 (14.4%)**	**4 (15.4%)**	**12 (14.1%)**	**0.01**
Condoms only	**30 (27.0%)**	**7 (26.9%)**	**23 (27.1%)**	
Oral contraceptive pills	**12 (10.8%)**	**3 (11.5%)**	**9 (10.6%)**	
Injectable	**25 (22.5%)**	**3 (11.5%)**	**22 (25.9%)**	
Implant	**10 (9.0%)**	**1 (3.8%)**	**9 (10.6%)**	
IUD	**10 (9.0%)**	**2 (7.7%)**	**8 (9.4%)**	
Tubal ligation	**5 (4.5%)**	**5 (19.2%)**	**0 (0%)**	
Other^2^	**3 (2.7%)**	**1 (3.8%)**	**2 (2.4%)**	
Frequency of vaginal washing				
Reports vaginal washing (yes/no)	**17 (15.3%)**	**0 (0.0%)**^3^	**17 (20.0%)**	**0.01**
Method of vaginal washing				
Water only	10 (58.8%)	0 (0%)^3^	10 (58.8%)	NA
Soap and water	9 (52.9%)	0 (0%)^3^	9 (52.9%)	NA
Other^4^	1 (5.9%)	0 (0%)^3^	1 (5.9%)	NA
Sexual history				
Frequency of vaginal sex in the past week	**2 (1, 4)**	**1 (0, 3.3)**	**3 (1, 4)**	**0.01**
Unprotected sex in the past week	39 (35.1%)	11 (42.3%)	28 (32.9%)	0.4
No sex in the past week	**21 (18.9%)**	**10 (38.5%)**	**11 (12.9%)**	**0.008**
Number of different sex partners in the past week	**1 (1, 3)**	**1 (1, 1)**	**1 (1, 3.75)**	**<0.001**
Exchange of money/goods for sex	**61 (55.0%)**	**1 (1.6%)**	**60 (70.6%)**	**<0.001**
Physical Examination				
Vulvovaginitis present^5^	10 (9%)	4 (15.4%)	6 (7.1%)	0.2
Laboratory data				
Gonorrhea^6^	0	0	0	NA
Chlamydia^6^	8 (7.2%)	1 (3.8%)	7 (8.2%)	0.7
HSV-2^7^	70 (63.1%)	13 (50.0%)	57 (67.1%)	0.1
Vulvovaginal candidiasis	36 (32.4%)	9 (34.6%)	27 (31.8%)	0.8
*Trichomonas vaginalis*	2 (1.8%)	0 (0.0%)	2 (2.4%)	1.0
Cervicitis^8^	**17 (15.3%)**	**15 (57.7%)**	**2 (2.4%)**	**<0.001**
BV by Amsel’s criteria	**33 (29.7%)**	**13 (50.0%)**	**20 (23.5%)**	**0.01**
Nugent score 0–3	50 (45.0%)	12 (46.2%)	38 (44.7%)	0.7
Nugent score 4–6	19 (17.1%)	3 (11.5%)	16 (18.8%)	
Nugent score 7–10	42 (37.8%)	11 (42.3%)	31 (36.5%)	
Baseline Detection of Bacteria >LLD^9^				
*Lactobacillus crispatus*	28 (25.7%)	7 (26.9%)	21 (25.3%)	1.0
*Lactobacillus jensenii*	**28 (25.7%)**	**12 (46.2%)**	**16 (19.3%)**	**0.01**
*Lactobacillus iners*	97 (89.0%)	25 (96.2%)	72 (86.7%)	0.3
BVAB1	**25 (22.9%)**	**15 (57.7%)**	**10 (12.0%)**	**<0.001**
BVAB2	50 (45.9%)	14 (53.8%)	36 (43.4%)	0.4
*Mageeibacillus indolicus*	33 (30.3%)	10 (38.5%)	23 (27.7%)	0.3
*Atopobium vaginae*	82 (75.2%)	21 (80.8%)	61 (73.5%)	0.6
*Leptotrichia/Sneathia* species	77 (70.6%)	18 (69.2%)	59 (71.1%)	1.0
*Megasphaera* species	52 (47.7%)	16 (61.5%)	36 (43.4%)	0.1
*Gardnerella vaginalis*	98 (89.9%)	24 (92.3%)	74 (89.2%)	1.0
Baseline Detection of Bacteria >ROC cutoff^9^				
*Lactobacillus crispatus*	28 (25.7%)	7 (26.9%)	21 (25.3%)	1.0
*Lactobacillus jensenii*	**27 (24.8%)**	**12 (46.2%)**	**15 (18.1%)**	**0.008**
*Lactobacillus iners*	83 (76.1%)	23 (88.5%)	60 (72.3%)	0.1
BVAB1	**15 (13.8%)**	**8 (30.8%)**	**7 (8.4%)**	**0.008**
BVAB2	39 (35.8%)	11 (42.3%)	28 (33.7%)	0.5
*Mageeibacillus indolicus*	20 (18.3%)	7 (26.9%)	13 (15.7%)	0.2
*Atopobium vaginae*	56 (51.4%)	14 (53.8%)	42 (50.6%)	0.8
*Leptotrichia/Sneathia* species	33 (30.3%)	7 (26.9%)	26 (31.3%)	0.8
*Megasphaera* species	39 (35.8%)	9 (34.6%)	30 (36.1%)	1.0
*Gardnerella vaginalis*	39 (35.8%)	9 (34.6%)	30 (36.1%)	1.0

Baseline data on age, marital status, contraceptive use, exchange of goods for sex, vaginal washing practices, and prevalence of *N*. *gonorrhoeae*, *C*. *trachomatis*, and herpes simplex virus 2 (HSV-2) infection were collected at enrollment. The remaining data, including microbiologic data, were collected at the first examination visit after enrollment (see methods). Data are presented as N (%) or median (interquartile range). Abbreviations: LLD, lower limit of detection; ROC, receiver operating curve; BVAB, bacterial vaginosis associated bacterium; HSV-2, herpes simplex virus 2; IUD, intrauterine device.

^1^Women were asked about race and Hispanic ethnicity separately. One woman reported both black race and Hispanic ethnicity.

^2^Other included fertility awareness method, herbal pill, and withdrawal.

^3^Although none of the US women reported vaginal washing at the baseline visit for this study, 7/26 (26.9%) reported vaginal washing at one or more visits. Among the total 16 visits at which US women reported vaginal washing, 1 (6.3%) reported using water only, 5 (31.3%) reported using water with soap, 2 (12.5%) reported using vinegar and water, and 8 (50%) reported using store-bought products.

^4^Other included use of salt and water.

^5^Vulvovaginitis was defined as the presence of at least 1 sign (tenderness, abnormal discharge, erythema, edema and rash as determined by a clinician on examination) and 1 symptom (self-reported vulvovaginal itching or pain) or the presence of two signs in the absence of symptoms.

^6^*Neisseria gonorrhoeae* and *Chlamydia trachomatis* positivity was determined by NAAT testing.

^7^HSV-2 serologic positivity was defined as optical density >2.1 in Kenyan women and positive vs. negative in US women.

^8^Cervicitis was defined as >30 polymorphonuclear cells per high-powered field.

^9^Two Kenyan women with longitudinal microbiota data did not have baseline microbiota data.

In the full cohort, there were no statistically significant differences in detection of bacteria above the LLD or ROC cutoffs at vaginal washing versus non-vaginal washing visits in either unadjusted analysis or analysis adjusted for age, HSV-2 serostatus, unprotected sex, and menstrual phase in the past week (Table 2). Testing for effect modification highlighted significant differences in the association between vaginal washing and detection of bacteria in US compared to Kenyan women for *M*. *indolicus* (P = 0.01) and *Megasphaera* species (p = 0.01). There was also some evidence suggesting effect modification for BVAB1 (p = 0.07), although this was not statistically significant at the α = 0.05 level. Thus, all analyses were subsequently stratified by country.

**Table 2:** Detection of bacteria (>LLD and >ROC cut-offs) at visits when women did versus did not report vaginal washing.

Lower limit of detection cutoff	Proportion of Visits with Organism Detected		Unadjusted Analysis^3^	Adjusted Analysis^4^
Organism	Non-washing visits >LLD (N = 537)^1^	Washing visits >LLD (N = 93)^1^	RR (95% CI), p-value^2^	RR (95% CI), p-value^2^
*Lactobacillus crispatus*	157 (29.2%)	17 (18.3%)	0.62 (0.38, 1.01), p = 0.06	0.64 (00.38, 1.06), p = 0.2
*Lactobacillus jensenii*	141 (26.3%)	20 (21.5%)	0.84 (0.51, 1.41), p = 0.5	0.78 (0.46, 1.31), p = 0.3
*Lactobacillus iners*	471 (87.7%)	87 (93.5%)	1.08 (0.99, 1.16), p = 0.07	1.07 (0.98, 1.16), p = 0.1
BVAB1	125 (23.3%)	25 (26.9%)	1.36 (0.93, 1.97), p = 0.1	1.26 (0.84, 1.89), p = 0.3
BVAB2	223 (41.5%)	45 (48.4%)	1.20 (0.87, 1.66), p = 0.3	1.17 (0.85, 1.60), p = 0.4
*Mageeibacillus indolicus*	161 (30.0%)	38 (40.9%)	1.44 (0.95, 2.18), p = 0.09	1.32 (0.89, 1.96), p = 0.2
*Atopobium vaginae*	387 (72.1%)	72 (77.4%)	1.08 (0.90, 1.30), p = 0.4	1.06 (0.88, 1.27), p = 0.5
*Leptotrichia/Sneathia* species	323 (60.1%)	62 (66.7%)	1.12 (0.90, 1.39), p = 0.3	1.10 (0.89, 1.35), p = 0.4
*Megasphaera* species	234 (43.6%)	42 (46.2%)	1.09 (0.76, 1.57), p = 0.6	1.05 (0.75, 1.47), p = 0.8
*Gardnerella vaginalis*	488 (90.9%)	84 (90.3%)	1.00 (0.92, 1.08), p = 0.9	0.99 (0.91, 1.08), p = 0.8
**Receiver operating curve cutoff**				
*Lactobacillus crispatus*	157 (29.2%)	17 (18.3%)	0.62 (0.38, 1.01), p = 0.06	0.64 (0.38, 1.06), p = 0.08
*Lactobacillus jensenii*	137 (25.5%)	20 (21.5%)	0.87 (0.52, 1.46), p = 0.6	0.80 (0.47, 1.36), p = 0.4
*Lactobacillus iners*	378 (70.4%)	60 (64.5%)	0.93 (0.77, 1.13), p = 0.5	0.92 (0.75, 1.12), p = 0.4
*BVAB1*	80 (14.9%)	12 (12.9%)	1.00 (0.55, 1.81), p = 1.0	0.94 (0.51, 1.73), p = 0.8
*BVAB2*	175 (32.6%)	29 (31.2%)	1.00 (0.65, 1.54), p = 1.0	0.97 (0.63, 1.50), p = 0.9
*Mageeibacillus indolicus*	109 (20.3%)	19 (20.4%)	1.08 (0.55, 2.11), p = 0.8	1.00 (0.53, 1.90), p = 1.0
*Atopobium vaginae*	243 (45.3%)	41 (44.1%)	0.99 (0.72, 1.37), p = 1.0	0.94 (0.69, 1.28), p = 0.7
*Leptotrichia/Sneathia* species	152 (28.3%)	23 (24.7%)	0.88 (0.54, 1.42), p = 0.6	0.91 (0.57, 1.44), p = 0.7
*Megasphaera* species	173 (32.2%)	33 (35.5%)	1.14 (0.72, 1.79), p = 0.6	1.09 (0.71, 1.67), p = 0.7
*Gardnerella vaginalis*	183 (34.1%)	26 (28.0%)	0.84 (0.55, 1.27), p = 0.2	0.80 (0.53, 1.20), p = 0.3

Abbreviations: LLD, lower limit of detection; ROC, receiver operating curve; RR, relative risk; CI, confidence interval; BVAB, bacterial vaginosis associated bacterium.

^1^Data presented as number (%).

^2^Relative risks comparing washing visits to non-washing visits were calculated using generalized estimating equation models with a Poisson link, independent correlation structure and robust errors for the outcomes: 1) above the LLD and 2) above the ROC cutoff for the bacterial concentration that maximizes prediction of BV.

^3^Controlling for country.

^4^Controlling for country, age, HSV-2, unprotected sex, and phase of menstrual cycle.

**Fig 1:**
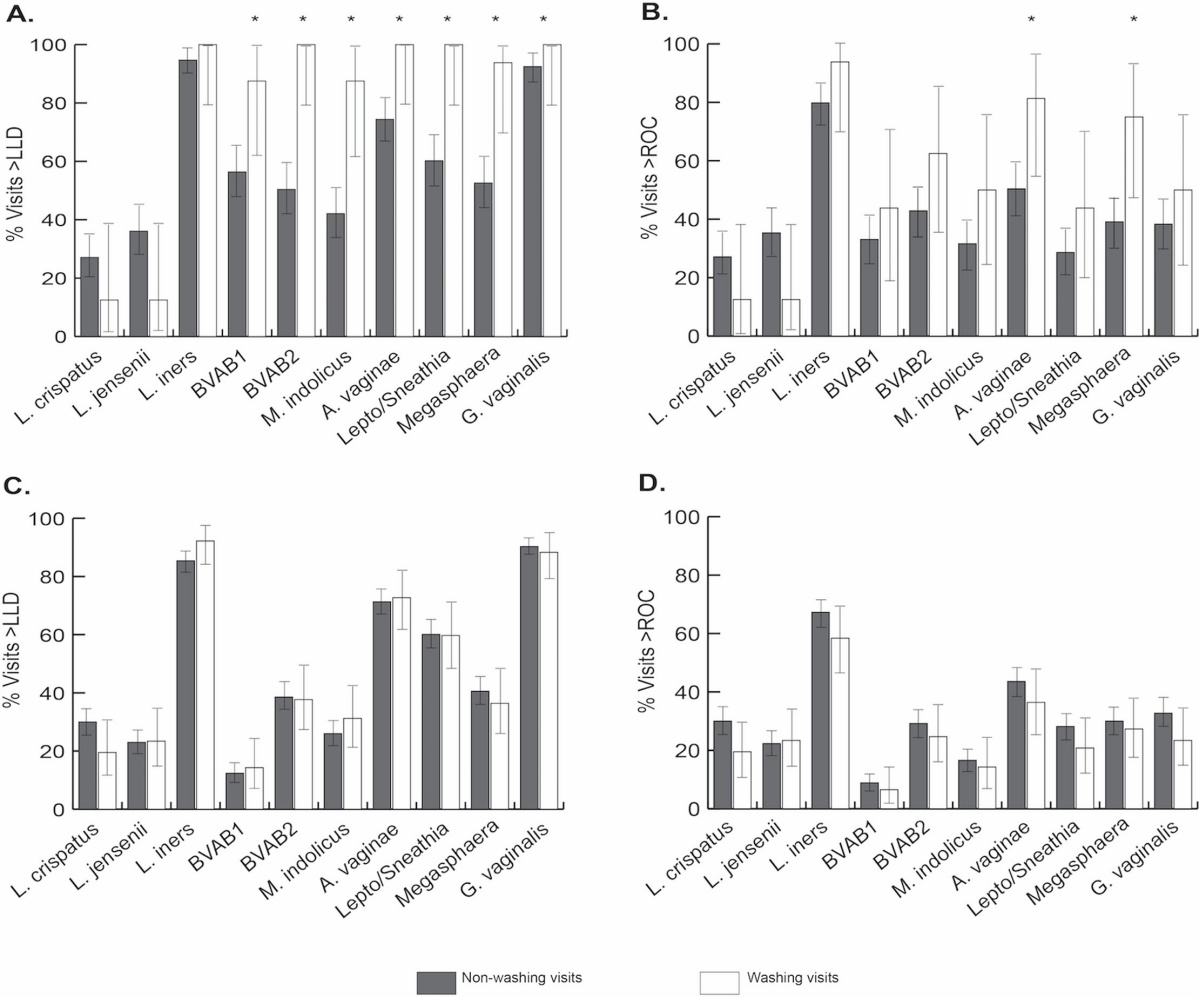
Comparison of bacterial taxa >LLD and >ROC cutoff at washing versus non-washing visits. The figure shows the proportion of vaginal washing visits versus non-vaginal washing visits at which concentrations of bacteria detected by 16S rRNA gene PCR were above the LLD cutoff (panel A, US; panel C, Kenya) and above the ROC cutoff predictive of BV (panel B, US; panel D, Kenya). Error bars represent 95% confidence intervals around the proportions. Asterisks (*) represent p-values that were <0.05 in the adjusted analysis.

In US women, vaginal washing was associated with a significantly higher likelihood of detection of BVAB1, BVAB2, *M*. *indolicus*, *A*. *vaginae*, *Leptotrichia/Sneathia* species, *Megasphaera* species, and *G*. *vaginalis* using the LLD cut-off (Fig 1A and Table 3). *Atopobium vaginae* and *Megasphaera* species were also significantly more likely to be detected at levels above the ROC cutoffs when vaginal washing was reported (Fig 1B and Table 3). The associations between vaginal washing and other bacterial taxa examined in the study were not substantially altered after adjustment for potential confounding factors. In addition, results were similar when BV status by Nugent score was added to the multivariable model (S1 Table). The US women were more likely to have BV based on Nugent score ≥7 versus <7 at vaginal washing visits compared to non-washing visits (10/16 [62.5%] vs. 57/132 [43.2%]; RR 1.45, 95%CI 0.92–2.29, p = 0.1), although this result was not statistically significant.

**Table 3:** Detection of bacteria (>LLD and >ROC cutoffs) for US participants at visits when women did versus did not report vaginal washing.

Lower limit of detection cutoff	Proportion of Visits with Taxa Detected		Unadjusted Analysis	Adjusted Analysis^3^
Organism	Non-washing visits >LLD (N = 133)^1^	Washing visits >LLD (N = 16)^1^	RR (95% CI), p-value^2^	RR (95% CI), p-value^2^
*Lactobacillus crispatus*	36 (27.1%)	2 (12.5%)	0.46 (0.13, 1.69), p = 0.2	1.09 (0.29, 4.07), p = 0.9
*Lactobacillus jensenii*	48 (36.1%)	2 (12.5%)	0.35 (0.05, 2.22), p = 0.3	0.47 (0.07, 3.22), p = 0.4
*Lactobacillus iners*	126 (94.7%)	16 (100%)	1.06 (0.96, 1.16), p = 0.2	1.02 (0.96, 1.08), p = 0.5
BVAB1	**75 (56.4%)**	**14 (87.5%)**	**1.55 (1.15, 2.09), p = 0.004**	1.30 (0.94, 1.78), p = 0.1
BVAB2	**67 (50.4%)**	**16 (100%)**	**1.99 (1.46, 2.71), p<0.001**	**1.91 (1.26, 2.90), p = 0.002**
*Mageeibacillus indolicus*	**56 (42.1%)**	**14 (87.5%)**	**2.08 (1.46, 2.96), p<0.001**	**1.87 (1.19, 2.94), p = 0.007**
*Atopobium vaginae*	**99 (74.4%)**	**16 (100%)**	**1.34 (1.13, 1.59), p = 0.001**	**1.32 (1.07, 1.63), p = 0.009**
*Leptotrichia/Sneathia* species	**80 (60.2%)**	**16 (100%)**	**1.66 (1.33, 2.09), p<0.001**	**1.50 (1.13, 1.99), p = 0.005**
*Megasphaera* species	**70 (52.6%)**	**15 (93.8%)**	**1.78 (1.34, 2.37), p<0.001**	**1.59 (1.12, 2.25), p = 0.01**
*Gardnerella vaginalis*	**123 (92.5%)**	**16 (100%)**	**1.08 (1.01, 1.16), p = 0.02**	1.09 (0.98, 1.21), p = 0.1
**Receiver operating curve cutoff**				
*Lactobacillus crispatus*	36 (27.1%)	2 (12.5%)	0.46 (0.13, 1.69), p = 0.2	1.09 (0.29, 4.07), p = 0.9
*Lactobacillus jensenii*	47 (35.3%)	2 (12.5%)	0.35 (0.06, 2.27), p = 0.3	0.48 (0.07, 3.32), p = 0.5
*Lactobacillus iners*	106 (79.6%)	15 (93.8%)	1.18 (1.00, 1.39), p = 0.05	1.15 (0.99, 1.33), p = 0.07
BVAB1	44 (33.1%)	7 (43.8%)	1.32 (0.67, 2.55), p = 0.4	1.23 (0.59, 2.59), p = 0.6
BVAB2	57 (42.9%)	10 (62.5%)	1.46 (0.92, 2.30), p = 0.1	1.42 (0.73, 2.74), p = 0.3
*Mageeibacillus indolicus*	42 (31.6%)	8 (50.0%)	1.58 (0.79, 3.17), p = 0.2	1.49 (0.67, 3.33), p = 0.3
*Atopobium vaginae*	**67 (50.4%)**	**13 (81.3%)**	**1.61 (1.08, 2.41), p = 0.02**	1.67 (0.97, 2.86), p = 0.07
*Leptotrichia/Sneathia* species	38 (28.6%)	7 (43.8%)	1.53 (0.83, 2.83), p = 0.2	1.65 (0.92, 2.96), p = 0.09
*Megasphaera* species	**52 (39.1%)**	**12 (75.0%)**	**1.92 (1.23, 3.00), p = 0.004**	**1.78 (1.07, 2.96), p = 0.03**
*Gardnerella vaginalis*	51 (38.3%)	8 (50.0%)	1.30 (0.77, 2.22), p = 0.3	1.03 (0.50, 2.09), p = 0.9

Abbreviations: LLD, lower limit of detection; ROC, receiver operating curve; RR, relative risk; CI, confidence interval, BVAB, bacterial vaginosis associated bacterium.

^1^Data presented as number (%).

^2^Relative risks comparing washing visits to non-washing visits were calculated using generalized estimating equation models with a Poisson link, independent correlation structure and robust errors for the outcomes: 1) above the LLD and 2) above the ROC cutoff for the bacterial concentration that maximizes prediction of BV.

^3^Controlling for age, HSV-2, unprotected sex, and phase of menstrual cycle.

**Fig 2:**
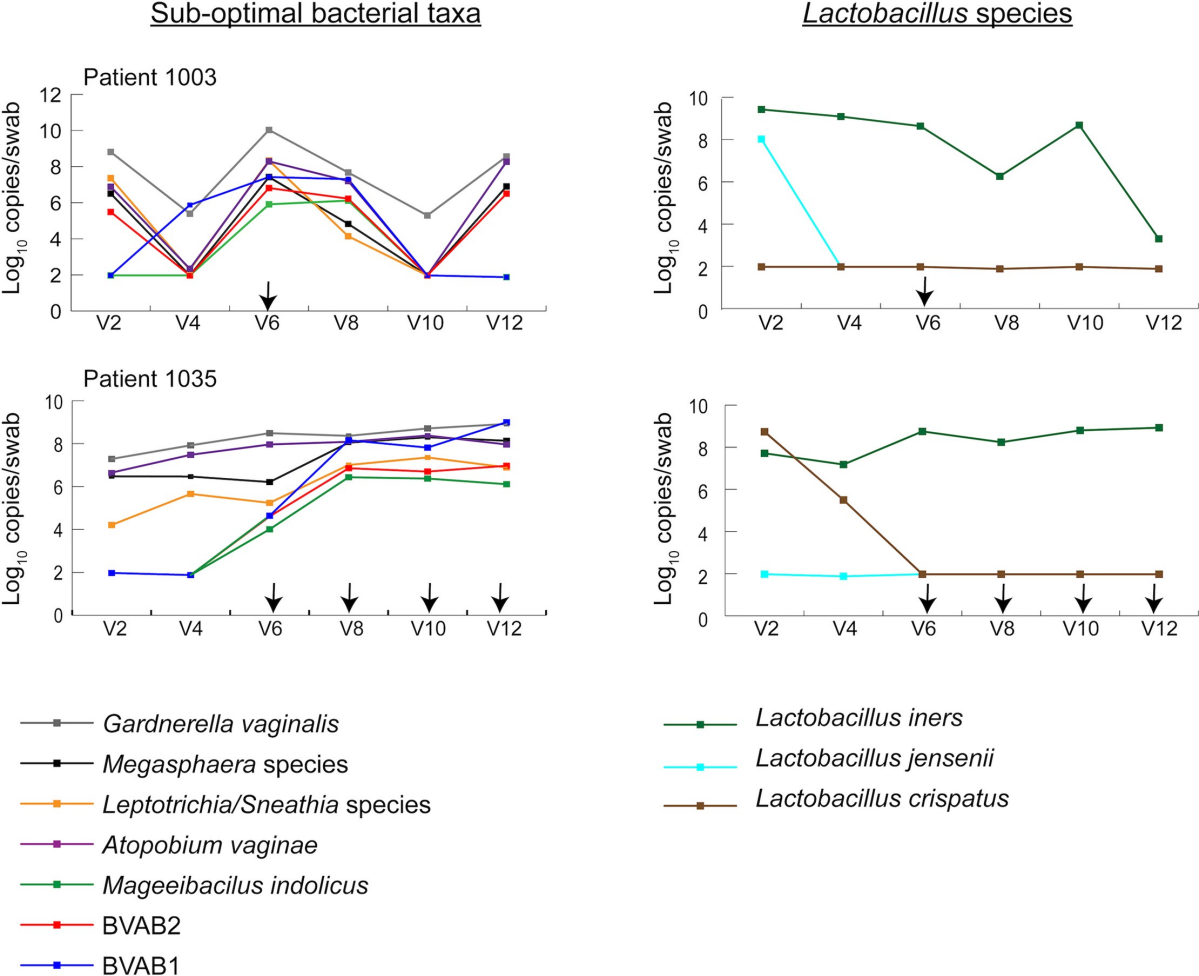
Changes in bacterial concentration over time in two US women who reported vaginal washing at some study visits. Log_10_ qPCR copies per vaginal swab of bacterial taxa were plotted versus study visit for two of the seven US women who reported vaginal washing. Sub-optimal bacterial taxa are plotted on the left, and *Lactobacillus* species are on the right. Visits at which vaginal washing was reported in the past month are marked with a black arrow.

To assess longitudinal trends in concentrations of vaginal bacteria among US women reporting vaginal washing, bacterial concentrations were plotted by visit number. In some women, higher concentrations of sub-optimal vaginal bacteria were evident at visits when vaginal washing was reported (for example, patient 1003 and patient 1035) (Fig 2). In other women, these shifts were less striking (S1 Fig).

In contrast to the US women, Kenyan women had no significant difference in concentrations of bacterial species >LLD cutoff or >ROC cutoff at vaginal washing versus non-vaginal washing visits (Fig 1C and 1D, and Table 4). These results were similar, and remained non-significant, with adjustment for BV diagnosed by Gram stain (S2 Table). Additionally, no significant difference was observed in BV by Nugent score ≥7 vs. <7 at vaginal washing versus non-vaginal washing visits (20/77 [26.0%] vs. 117/404 [29.0%]; RR 0.9, 95%CI 0.53–1.53, p = 0.7).

**Table 4:** Detection of bacteria (>LLD and >ROC cutoffs) for Kenyan participants at visits when women did versus did not report vaginal washing.

Lower limit of detection cutoff	Proportion of Visits with Taxa Detected		Unadjusted Analysis	Adjusted Analysis^3^
Organism	Non-washing visits >LLD (N = 404)^1^	Washing visits >LLD (N = 77)^1^	RR (95% CI), p-value^2^	RR (95% CI), p-value^2^
*Lactobacillus crispatus*	121 (30.0%)	15 (19.5%)	0.65 (0.39, 1.10), p = 0.1	0.70 (0.41, 1.19), p = 0.7
*Lactobacillus jensenii*	93 (23.0%)	18 (23.4%)	1.02 (0.60, 1.71), p = 1.0	0.98 (0.58, 1.65), p = 0.9
*Lactobacillus iners*	345 (85.4%)	71 (92.2%)	1.08 (0.98, 1.19), p = 0.1	1.07 (0.97, 1.18). p = 0.2
BVAB1	50 (12.4%)	11 (14.3%)	1.15 (0.56, 2.40), p = 0.7	0.95 (0.44, 2.02), p = 0.9
BVAB2	156 (38.6%)	29 (37.7%)	0.98 (0.64, 1.50), p = 0.9	0.93 (0.62, 1.40), p = 0.7
*Mageeibacillus indolicus*	105 (26.0%)	24 (31.2%)	1.20 (0.65, 2.21), p = 0.6	1.01 (0.60, 1.69), p = 1.0
*Atopobium vaginae*	288 (71.3%)	56 (72.7%)	1.02 (0.82, 1.28), p = 0.9	1.00 (0.80, 1.25), p = 1.0
*Leptotrichia/Sneathia* species	243 (60.1%)	46 (59.7%)	0.99 (0.76, 1.30), P = 1.0	0.95 (0.74, 1.22), p = 0.7
*Megasphaera* species	164 (40.6%)	28 (36.4%)	0.90 (0.55, 1.47), p = 0.7	0.86 (0.55, 1.35), p = 0.5
*Gardnerella vaginalis*	365 (90.3%)	68 (88.3%)	0.98 (0.88, 1.08), p = 0.7	0.97 (0.87, 1.08), p = 0.6
**Receiver operating curve cutoff**				
*Lactobacillus crispatus*	121 (30.0%)	15 (19.5%)	0.65 (0.39, 1.10), p = 0.1	0.70 (0.41, 1.19), p = 0.2
*Lactobacillus jensenii*	90 (22.3%)	18 (23.4%)	1.05 (0.62, 1.78), p = 0.9	1.02 (0.60, 1.74), p = 0.9
*Lactobacillus iners*	272 (67.3%)	45 (58.4%)	0.87 (0.68, 1.11), p = 0.3	0.84 (0.66, 1.07), p = 0.2
BVAB1	36 (8.9%)	5 (6.5%)	0.73 (0.28, 1.91), p = 0.5	0.53 (0.19, 1.44), p = 0.2
BVAB2	118 (29.2%)	19 (24.7%)	0.85 (0.46, 1.54), p = 0.6	0.82 (0.46, 1.48), p = 0.5
*Mageeibacillus indolicus*	67 (16.6%)	11 (14.3%)	0.86 (0.31, 2.41), p = 0.8	0.75 (0.29, 1.94), p = 0.5
*Atopobium vaginae*	176 (43.6%)	28 (36.4%)	0.84 (0.56, 1.25), p = 0.4	0.80 (0.55, 1.17), p = 0.3
*Leptotrichia/Sneathia* species	114 (28.2%)	16 (20.8%)	0.74 (0.40, 1.35), p = 0.3	0.75 (0.42, 1.34), p = 0.3
*Megasphaera* species	121 (30.0%)	21 (27.3%)	0.91 (0.49, 1.69), p = 0.8	0.85 (0.48, 1.48), p = 0.8
*Gardnerella vaginalis*	132 (32.7%)	18 (23.4%)	0.72 (0.41, 1.24), p = 0.2	0.68 (0.39, 1.18), p = 0.2

Abbreviations: LLD, lower limit of detection; ROC, receiver operating characteristic; RR, relative risk; CI, confidence interval; BVAB, bacterial vaginosis associated bacterium.

^1^Data presented as number (%).

^2^Relative risks comparing washing visits to non-washing visits were calculated using generalized estimating equation models with a Poisson link, independent correlation structure and robust errors for the outcomes: 1) above the LLD and 2) above the ROC cut-off for the bacterial concentration that maximizes prediction of BV.

^3^Controlling for age, HSV-2, unprotected sex, and phase of menstrual cycle.

## Discussion

In this exploratory analysis, vaginal washing was associated with increased detection and higher concentrations of several vaginal bacteria associated with vaginal dysbiosis and BV in US women, but not in Kenyan women. Specifically, BVAB1, BVAB2, *M*. *indolicus*, *A*. *vaginae*, *Leptotrichia/Sneathia* species, *Megasphaera* species, and *G*. *vaginali*s were detected with increased frequency in US women at vaginal washing visits. Additionally, *A*. *vaginae* and *Megasphaera* species were more likely to be found at concentrations associated with BV at vaginal washing visits in US women. These associations were similar when analyses were adjusted for the presence of BV, suggesting an association with individual bacterial detection and concentrations that is independent of the previously recognized association between vaginal washing and BV by Gram stain [6–8, 10].

There are several possible explanations for the difference in associations between vaginal washing and detection of specific bacteria in the US compared to Kenya. First, US women were more likely to report using commercial products at vaginal washing visits. It is possible that these products have a greater impact on the vaginal microbiota compared to water (with or without soap), which were used by the majority of Kenyan women. Second, although all women who reported vaginal washing also reported black race, the vaginal microbiome may also vary by geographic region and ethnicity [18, 25, 26]. The bacteria tested in this analysis were selected based on earlier studies that showed their association with BV in US women [14]. In Kenyan women, vaginal washing may have a greater impact on other bacterial taxa that were not tested in this study. Further studies conducted on samples from Kenyan women using broad range PCR and deep sequencing, as well as with qPCR targeting additional bacterial taxa, may help to address this question. Third, baseline differences in bacterial taxa may have increased the likelihood that vaginal washing would lead to disruption of the vaginal microbiota. Interestingly, *L*. *jensenii* was detected with increased frequency in US versus Kenyan women, and has been associated with increased vaginal microbiota instability in pregnant women [27]. Fourth, US and Kenyan women may differ in their accuracy of reporting intravaginal practices. For example, if underreporting of vaginal washing was more common in Kenyan participants, the findings in the Kenyan cohort would be biased more strongly toward finding no association. Finally, it is possible that the observed associations in US women occurred by chance, or that this analysis failed to detect true differences associated with washing in Kenyan women. Regardless of what factors led to the difference in results between these two countries, these data highlight the fact that vaginal washing encompasses a wide range of practices that may have distinct effects on the microbiota in different populations. It follows that caution should be used when trying to generalize the results of vaginal washing studies across different populations and geographic regions.

Within the US population, it is important to consider the possible explanations for the observed associations between vaginal washing and detection and concentrations of BV-related bacteria. Washing practices may impact the vaginal ecosystem by selecting for sub-optimal bacteria. Alternatively, vaginal washing may be a response to symptoms caused by particular bacteria [22]. It is also possible that both mechanisms act synergistically, perpetuating a harmful cycle in which women wash to reduce odor and discharge, only to develop more symptoms, resulting in more washing [10, 20, 28]. Adjustment for phase of menstrual cycle had little impact on the results, suggesting that the associations presented here were not due to cyclic variation in the microbiome or washing practices [20]. Overall, the direction of these effects cannot be determined through observational data, and will require interventional studies that include cessation of vaginal washing.

This study had a number of strengths. First, identical procedures were applied to geographically distinct populations with different vaginal washing practices, allowing a direct test of the hypothesis that the association of vaginal washing with the concentrations of individual vaginal bacteria would vary across populations. Second, this study is unique in using taxon-specific qPCR to evaluate the association between vaginal washing and concentrations of individual vaginal bacteria. The use of taxon-directed qPCR is an important contribution, as adverse health outcomes have been associated with concentrations of specific bacteria [12, 13]. Third, results remained significant after additional adjustment for diagnosis of BV by Nugent score, suggesting that the association between vaginal washing and vaginal bacterial concentrations was independent of the well characterized association between vaginal washing and BV [6–8, 10]. Finally, retention rates in the parent study were high, minimizing the risk of bias due to attrition.

The findings from this study should be interpreted in the context of several limitations. This was an exploratory secondary analysis without adjustment for multiple comparisons, and the results should be considered as hypothesis generating. Although the findings in the US population were significant, the results are based on only 26 women, 7 of whom contributed a total of 16 visits where vaginal washing was reported. While the small sample size does not necessarily introduce bias, it does lead to large confidence intervals around the point estimates of the association between vaginal washing and individual bacterial concentrations. Larger studies will be helpful for both confirming the findings and generating more precise effect estimates. Additionally, use of a monthly vaginal suppository may have altered the vaginal microbiome. However, placebo suppositories were only used for five nights, and it was recommended that they be used at the beginning of each month of follow-up. Because vaginal samples were collected at the end of each monthly follow-up interval, the majority were likely collected more than three weeks after use of the placebo product. In addition, trial participants were asked to use the study product in the same way regardless of vaginal washing practices, so this should not have altered our ability to compare between vaginal washing and non-vaginal washing visits.

In summary, this analysis demonstrated associations between vaginal washing and the presence and concentrations of individual vaginal bacteria in US women, but not in Kenyan women. These findings have several implications for future research to improve women’s health. First, the effect of vaginal washing may vary regionally and between ethnic populations with distinct vaginal washing practices. Future studies in multiple geographic regions may be required to gain a holistic understanding of the range of potential impacts of vaginal washing on vaginal health. Second, these results suggest that the effect of vaginal washing on individual vaginal bacterial taxa may be independent of the impact of these practices on the diagnosis of BV by Gram stain. It follows that use of molecular methods will be important in future vaginal washing studies to capture potentially important effects on bacteria associated with adverse health outcomes. Randomized trials of vaginal washing cessation interventions will be essential for establishing or disproving causal relationships between vaginal washing and the vaginal microbiota, and for determining whether refraining from vaginal washing can restore optimal vaginal microbiota.

## Supporting information

S1 Table Detection of bacteria (>LLD and >ROC cut-offs) for US participants at visits when women did versus did not report vaginal washing, including adjustment for bacterial vaginosis. Including diagnosis of bacterial vaginosis in the adjusted analysis did not significantly alter the associations between vaginal washing and detection of bacterial taxa in US women.
(DOCX)

S2 Table Detection of bacteria (>LLD and >ROC cut-offs) for Kenyan participants at visits when women did versus did not report vaginal washing, including adjustment for bacterial vaginosis. Including diagnosis of bacterial vaginosis in the adjusted analysis did not significantly alter the associations between vaginal washing and detection of bacterial taxa in Kenyan women.
(DOCX)

S1 Fig Changes in bacterial concentration over time in US women who reported vaginal washing. Log_10_ copies per vaginal swab of bacterial taxa were plotted versus study visit. Sub-optimal bacterial taxa are plotted on the left, and *Lactobacillus* species are on the right. Study visits at which vaginal washing was reported are marked with a black arrow.
(TIFF)
