# Supplementary material for: Association between vaginal washing and vaginal bacterial concentrations
Source: PLoS One. 2019 Jan 24;14(1):e0210825. doi: 10.1371/journal.pone.0210825 (PMC6345501; doi:10.1371/journal.pone.0210825)
Supplement: S1 Table — Including diagnosis of bacterial vaginosis in the adjusted analysis did not significantly alter the associations between vaginal washing and detection of bacterial taxa in US women. (DOCX) [file pone.0210825.s001.docx]

**S1 Table**: Detection of bacteria (>LLD and >ROC cut-offs) for US participants at visits when women did versus did not report vaginal washing including adjustment for bacterial vaginosis

| **Lower limit of detection cutoff**  **Organism** | **Proportion of Visits with Taxa Detected** | | **Unadjusted Analysis** | **Adjusted Analysis^3^** |
| --- | --- | --- | --- | --- |
|  | Non-washing visits >LLD (N=133)^1^ | Washing visits >LLD  (N=16)^1^ | RR (95% CI), p-value^2^ | RR (95% CI), p-value^2^ |
| *Lactobacillus crispatus* | 36 (27.1%) | 2 (12.5%) | 0.46 (0.13, 1.69), p=0.2 | 0.57 (0.20, 1.60), p=0.3 |
| *Lactobacillus jensenii* | 48 (36.1%) | 2 (12.5%) | 0.35 (0.05, 2.22), p=0.3 | 0.45 (0.07, 3.06), p=0.4 |
| *Lactobacillus iners* | 126 (94.7%) | 16 (100%) | 1.06 (0.96, 1.16), p=0.2 | 1.06 (0.96, 1.18), p=0.3 |
| BVAB1 | 75 (56.4%) | 14 (87.5%) | 1.55 (1.15, 2.09), p=0.004 | 1.37 (1.07, 1.76), p=0.01 |
| BVAB2 | 67 (50.4%) | 16 (100%) | 1.99 (1.46, 2.71), p<0.001 | 1.68 (1.30, 2.17), p<0.001 |
| *Mageeibacillus indolicus* | 56 (42.1%) | 14 (87.5%) | 2.08 (1.46, 2.96), p<0.001 | 1.77 (1.28, 2.43), p<0.001 |
| *Atopobium vaginae* | 99 (74.4%) | 16 (100%) | 1.34 (1.13, 1.59), p=0.001 | 1.23 (1.09, 1.38), p=0.001 |
| *Leptotrichia/Sneathia* species | 80 (60.2%) | 16 (100%) | 1.66 (1.33, 2.09), p<0.001 | 1.47 (1.23, 1.76), p<0.001 |
| *Megasphaera* species | 70 (52.6%) | 15 (93.8%) | 1.78 (1.34, 2.37), p<0.001 | 1.49 (1.12, 1.98), p=0.006 |
| *Gardnerella vaginalis* | 123 (92.5%) | 16 (100%) | 1.08 (1.01, 1.16), p=0.02 | 1.06 (1.00, 1.11), p=0.03 |
| **Receiver operating curve cutoff** | | | | |
| *Lactobacillus crispatus* | 36 (27.1%) | 2 (12.5%) | 0.46 (0.13, 1.69), p=0.2 | 0.57 (0.20, 1.60), p=0.3 |
| *Lactobacillus jensenii* | 47 (35.3%) | 2 (12.5%) | 0.35 (0.06, 2.27), p=0.3 | 0.45 (0.07, 3.10), p=0.4 |
| *Lactobacillus iners* | 106 (79.6%) | 15 (93.8%) | 1.18 (1.00, 1.39), p=0.05 | 1.22 (1.02, 1.45), p=0.03 |
| BVAB1 | 44 (33.1%) | 7 (43.8%) | 1.32 (0.67, 2.55), p=0.4 | 0.94 (0.67, 1.32), p=0.7 |
| BVAB2 | 57 (42.9%) | 10 (62.5%) | 1.46 (0.92, 2.30), p=0.1 | 1.10 (0.76, 1.59), p=0.6 |
| *Mageeibacillus indolicus* | 42 (31.6%) | 8 (50.0%) | 1.58 (0.79, 3.17), p=0.2 | 1.21 (0.67, 2.18), p=0.5 |
| *Atopobium vaginae* | 67 (50.4%) | 13 (81.3%) | 1.61 (1.08, 2.41), p=0.02 | 1.33 (0.96, 1.84), p=0.09 |
| *Leptotrichia/Sneathia* species | 38 (28.6%) | 7 (43.8%) | 1.53 (0.83, 2.83), p=0.2 | 1.33 (0.70, 2.52), p=0.4 |
| *Megasphaera* species | 52 (39.1%) | 12 (75.0%) | 1.92 (1.23, 3.00), p=0.004 | 1.54 (1.00, 2.37), p=0.05 |
| *Gardnerella vaginalis* | 51 (38.3%) | 8 (50.0%) | 1.30 (0.77, 2.22), p=0.3 | 1.04 (0.66, 1.63), p=0.9 |

**S1 Table:** Abbreviations: LLD, lower limit of detection; ROC, receiver operator curve; RR, relative risk; CI, confidence interval*,* BVAB, bacterial vaginosis associated bacteria.

^1^Data presented as number (%).

^2^Relative risks comparing washing visits to non-washing visits were calculated using generalized estimating equation models with a Poisson link, independent correlation structure and robust errors for the outcomes: 1) above the LLD and 2) above the ROC cut-off for the bacterial concentration that maximizes prediction of BV.

^3^Controlling for age, HSV-2, unprotected sex, bacterial vaginosis
