# Supplementary material for: Association between vaginal washing and vaginal bacterial concentrations
Source: PLoS One. 2019 Jan 24;14(1):e0210825. doi: 10.1371/journal.pone.0210825 (PMC6345501; doi:10.1371/journal.pone.0210825)
Supplement: S2 Table — Including diagnosis of bacterial vaginosis in the adjusted analysis did not significantly alter the associations between vaginal washing and detection of bacterial taxa in Kenyan women. (DOCX) [file pone.0210825.s002.docx]

**S2 Table:** Detection of bacteria (>LLD and >ROC cut-offs) for Kenyan participants at visits when women did versus did not report vaginal washing, including adjustment for bacterial vaginosis

| **Lower limit of detection cutoff**  **Organism** | **Proportion of Visits with Taxa Detected** | | **Unadjusted Analysis** | **Adjusted Analysis^3^** |
| --- | --- | --- | --- | --- |
|  | Non-washing visits >LLD (N=404)^1^ | Washing visits >LLD (N=77)^1^ | RR (95% CI), p-value^2^ | RR (95% CI), p-value^2^ |
| *Lactobacillus crispatus* | 121 (30.0%) | 15 (19.5%) | 0.65 (0.39, 1.10), p=0.1 | 0.66 (0.39, 1.11), p=0.1 |
| *Lactobacillus jensenii* | 93 (23.0%) | 18 (23.4%) | 1.02 (0.60, 1.71), p=1.0 | 0.93 (0.58, 1.49), p=0.8 |
| *Lactobacillus iners* | 345 (85.4%) | 71 (92.2%) | 1.08 (0.98, 1.19), p=0.1 | 1.08 (0.97, 1.19), p=0.2 |
| BVAB1 | 50 (12.4%) | 11 (14.3%) | 1.15 (0.56, 2.40), p=0.7 | 1.01 (0.49, 2.09), p=1.0 |
| BVAB2 | 156 (38.6%) | 29 (37.7%) | 0.98 (0.64, 1.50), p=0.9 | 0.99 (0.73, 1.34), p=0.9 |
| *Mageeibacillus indolicus* | 105 (26.0%) | 24 (31.2%) | 1.20 (0.65, 2.21), p=0.6 | 1.08 (0.75, 1.56), p=0.7 |
| *Atopobium vaginae* | 288 (71.3%) | 56 (72.7%) | 1.02 (0.82, 1.28), p=0.9 | 1.02 (0.84, 1.22), p=0.9 |
| *Leptotrichia/Sneathia* species | 243 (60.1%) | 46 (59.7%) | 0.99 (0.76, 1.30), P=1.0 | 1.00 (0.81, 1.23), p=1.0 |
| *Megasphaera* species | 164 (40.6%) | 28 (36.4%) | 0.90 (0.55, 1.47), p=0.7 | 0.90 (0.62, 1.28), p=0.9 |
| *Gardnerella vaginalis* | 365 (90.3%) | 68 (88.3%) | 0.98 (0.88, 1.08), p=0.7 | 0.98 (0.89, 1.08), p=0.7 |
| **Receiver operating curve cutoff** | | | | |
| *Lactobacillus crispatus* | 121 (30.0%) | 15 (19.5%) | 0.65 (0.39, 1.10), p=0.1 | 0.66 (0.39, 1.11), p=0.7 |
| *Lactobacillus jensenii* | 90 (22.3%) | 18 (23.4%) | 1.05 (0.62, 1.78), p=0.9 | 0.96 (0.60, 1.55), p=0.9 |
| *Lactobacillus iners* | 272 (67.3%) | 45 (58.4%) | 0.87 (0.68, 1.11), p=0.3 | 0.85 (0.66, 1.09), p=0.2 |
| BVAB1 | 36 (8.9%) | 5 (6.5%) | 0.73 (0.28, 1.91), p=0.5 | 0.54 (0.21, 1.39), p=0.2 |
| BVAB2 | 118 (29.2%) | 19 (24.7%) | 0.85 (0.46, 1.54), p=0.6 | 0.89 (0.60, 1.32), p=0.6 |
| *Mageeibacillus indolicus* | 67 (16.6%) | 11 (14.3%) | 0.86 (0.31, 2.41), p=0.8 | 0.80 (0.39, 1.66), p=0.6 |
| *Atopobium vaginae* | 176 (43.6%) | 28 (36.4%) | 0.84 (0.56, 1.25), p=0.4 | 0.84 (0.64, 1.10), p=0.2 |
| *Leptotrichia/Sneathia* species | 114 (28.2%) | 16 (20.8%) | 0.74 (0.40, 1.35), p=0.3 | 0.81 (0.51, 1.27), p=0.4 |
| *Megasphaera* species | 121 (30.0%) | 21 (27.3%) | 0.91 (0.49, 1.69), p=0.8 | 0.91 (0.61, 1.35), p=0.6 |
| *Gardnerella vaginalis* | 132 (32.7%) | 18 (23.4%) | 0.72 (0.41, 1.24), p=0.2 | 0.74 (0.50, 1.11), p=0.2 |

**S2 Table**: Abbreviations: LLD, lower limit of detection; ROC, receiver operator characteristic; RR, relative risk; CI, confidence interval; BVAB, bacterial vaginosis associated bacteria.

^1^Data presented as number (%).

^2^Relative risks comparing washing visits to non-washing visits were calculated using generalized estimating equation models with a Poisson link, independent correlation structure and robust errors for the outcomes: 1) above the LLD and 2) above the ROC cut-off for the bacterial concentration that maximizes prediction of BV.

^3^Controlling for age, HSV-2, unprotected sex, bacterial vaginosis
