# Supplementary figures and images for: Association between vaginal washing and vaginal bacterial concentrations
Source: PLoS One. 2019 Jan 24;14(1):e0210825. doi: 10.1371/journal.pone.0210825 (PMC6345501; doi:10.1371/journal.pone.0210825)

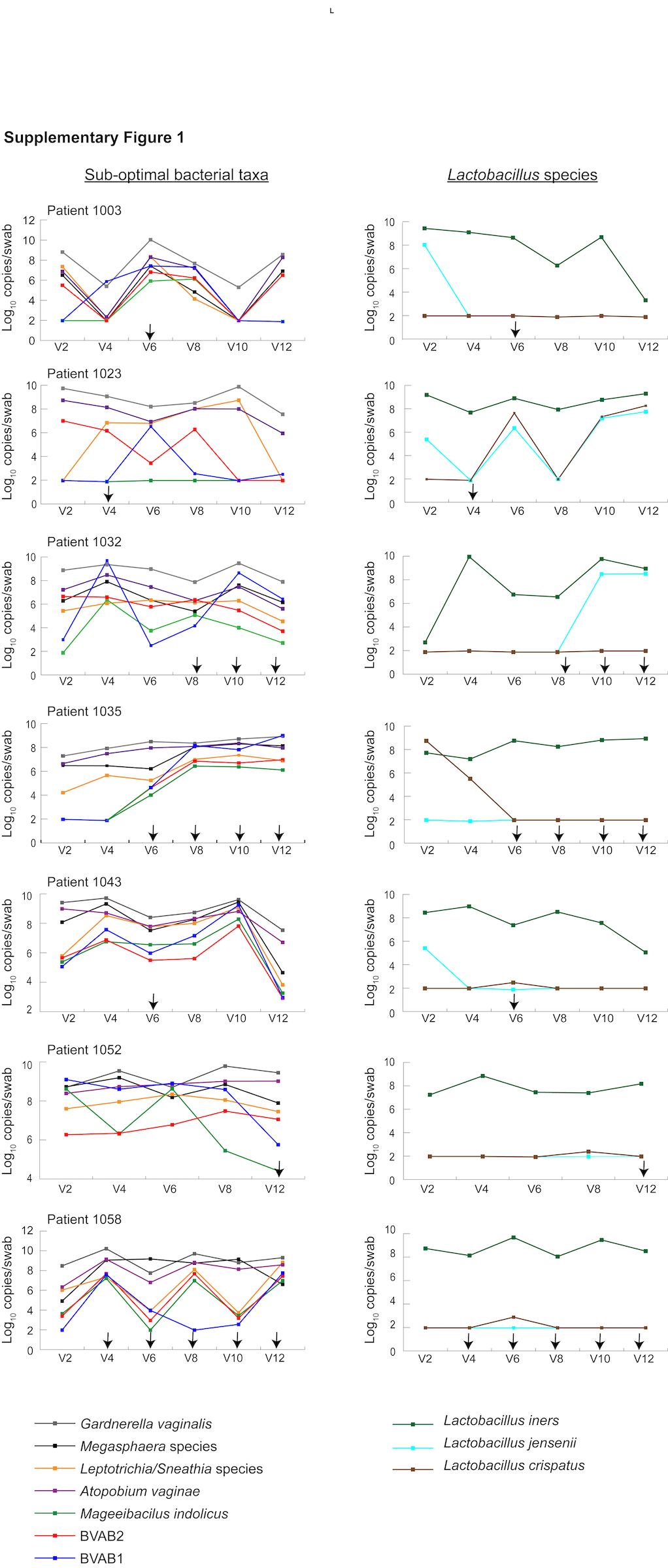

Supplement: S1 Fig — Log10 copies per vaginal swab of bacterial taxa were plotted versus study visit. Sub-optimal bacterial taxa are plotted on the left, and Lactobacillus species are on the right. Study visits at which vaginal washing was reported are marked with a black arrow. (TIFF) [file pone.0210825.s003.tiff]
